# Clinic of Prof. Garretson

**Published:** 1885-11

**Authors:** Robert S. Ivy


### HOSPITAL OF ORAL SURGERY, PHILADELPHIA.

### CLINIC OF PROF. GARRETSON.

REPORTED FOR THE INDEPENDENT PRACTITIONER BY ROBERT S. IVY, D. D. S.

#### EPULO-FIBROMA IN A PREGNANT WOMAN—OPERATION.

The patient, aged thirty-five, had been pregnant six months. The growth, for the treatment of which the woman had presented herself, was about the size of a hickory nut and occupied a position upon the right side of the upper jaw, enveloping a portion of the crowns of the lateral incisor, cuspid and bicuspid teeth. Attention was particularly called to a distinction to be recognized between the proliferations of gingivitis as occasionally met with in child-bearing women, and true neoplasms. These distinctions were clinically discussed, and a diagnosis of the case arrived at by exclusion, the tumor being pronounced an epulo-fibroma. The rapid development of the growth and the known tendency of the condition to malignant degeneration decided Professor Garretson to take the risks of radical extirpation, and accordingly the patient was at once etherized, and the tumor, teeth, and involved portion of the bone removed in a common section made by the circular saw.

It is the experience of the clinic that sections of the jaw bones made by a firm and rapidly revolving circular saw give little shock. In the present instance the patient had no occasion to go to bed, and after two or three days went about her household duties as usual. Prof. Garretson held that the risks of an abortion were less than the risks of waiting on the neoplasm.

OSTEO-SARCOMA. REMOVAL OF UPPER JAW, OF THE TURBINATED, NASAL AND LACHRYMAL BONES, AND A PORTION OF THE ETHMOID.

This was an operation of uncommon severity, and is not known to the writer as having ever before been practiced. The operation performed before the class was a success to such an uncommon extent, that the man was permitted to leave the ward after fourteen days, to return to his home and the care of the family physician.

Uncovering of the parts involved was secured by the use of two incisions, the first beginning over the region of the frontal sinus and descending down along the side of the nose until reaching the lip, through the median line of which it passed; the second commenced at the inner canthus of the eye and passed outward to the malar bone. Flaps, thus secured, were raised from the nasal and jaw regions.

The removal of the bones involved was effected by the use of revolving saws, burs and lion forceps. As the maxilla was concerned, its ablation was secured by cutting the malo-maxillary, the naso-maxillary, the palato-maxillary, and spheno-maxillary articulations, and then lifting it from its bed by means of lion forceps.

The hemorrhage, which was necessarily very profuse, was controlled by use of ligatures and compresses. The cavity formed by the removal of the diseased parts was subsequently packed with phenated sponges, having strong ligatures attached to permit of withdrawal. A succeeding step was to replace the flap, its edges being held in apposition by sutures, the incision at the lip being drawn together by pins, after the manner of a hare-lip operation. A section of the tumor removed, when examined under the microscope, was seen to contain spindle-shaped and giant cells, the latter resembling osteoclasts, both of these being characteristic of the myeloid sarcoma. Three days after the operation, the pins holding the lip together and many of the stitches approximating the wound were removed, and the patient was able to walk about the ward unaided. One week later he was shown to the class, all the stitches having been taken away, the wound having absolutely united by first intention, while very slight disfigurement was observable. The eye remained uninjured, and had all its natural movements.

OSTEO-FIBROMA. DISARTICULATION OF THE LOWER JAW.

This was another of the many extensive performances practiced at the oral clinic. Patient, a vigorous man, sanguine temperament, aged thirty-five. The growth was of two years' standing, with the general health unimpaired. The deformity was very great, the tumor measuring four inches in circumference. The operation was commenced, like the former, with great regard to the extent of anæsthesia produced, Professor Garretson asserting that it needed care alone in this direction to render unnecessary the preliminary performance of tracheotomy, as recommended and practiced in German surgery; "he had performed the operation too often," he said, "to fear drowning a patient in his own blood."

The disarticulation was commenced by an incision beginning in a division of the lower lip at the exact median line, and which was carried around and beneath the jaw until it terminated at the ear. The flap thus relieved was dissected from the face of the tumor, and the bone being divided, by means of a circular saw, at the symphysis, was turned out and disarticulated. To avoid wounding the internal carotid and internal maxillary arteries, the professor used the knife very little about the region of the head of the bone, depending here upon the aid of a chisel-shaped gouge. A stout tenotome was employed to cut away the attachments of the pterygoid and temporal muscles.

Dr. Garretson is ably seconded in all his clinical operations by his chief of clinic, Dr. Cryer, the two having so long operated together as to appreciate urgent and quick wants, without the necessity for speaking a word.

